# Downregulated miR-195 Detected in Preeclamptic Placenta Affects Trophoblast Cell Invasion via Modulating ActRIIA Expression

**DOI:** 10.1371/journal.pone.0038875

**Published:** 2012-06-19

**Authors:** Yang Bai, Weiwei Yang, Hui-xia Yang, Qinping Liao, Gang Ye, Guodong Fu, Lei Ji, Peng Xu, Hao Wang, Yu-xia Li, Chun Peng, Yan-ling Wang

**Affiliations:** 1 State Key Laboratory of Reproductive Biology, Institute of Zoology, Chinese Academy of Sciences, Beijing, China; 2 Department of Obstetrics and Gynecology, Peking University First Hospital, Beijing, China; 3 Department of Biology, York University, Toronto, Ontario, Canada; 4 Graduate School of Chinese Academy of Sciences, Beijing, China; Fudan University, China

## Abstract

**Background:**

Preeclampsia (PE) is a pregnancy-specific syndrome manifested by on-set of hypertension and proteinuria after 20 weeks of gestation. Abnormal placenta development has been generally accepted as initial cause of the disorder. Recently, miR-195 was found to be down-regulated in preeclamptic placentas compared with normal pregnant ones, indicating possible association of this small molecule with placental pathology of preeclampsia. By far the function of miR-195 in the development of placenta remains unknown.

**Methodology/Principal Findings:**

Bioinformatic assay predicted ActRIIA as one of the targets for miR-195. By using Real-time PCR, Western blotting and Dual Luciferase Assay, we validated that ActRIIA was the direct target of miR-195 in human trophoblast cells. Transwell insert invasion assay showed that miR-195 could promote cell invasion in trophoblast cell line, HTR8/SVneo cells, and the effect could be abrogated by overexpressed ActRIIA. In preeclamptic placenta tissues, pri-miR-195 and mature miR-195 expressions were down-regulated, whereas ActRIIA level appeared to be increased when compared with that in gestational-week-matched normal placentas.

**Conclusions/Significance:**

This is the first report on the function of miR-195 in human placental trophoblast cells which reveals an invasion-promoting effect of the small RNA via repressing ActRIIA. Aberrant expression of miR-195 may contribute to the occurrence of preeclampsia through interfering with Activin/Nodal signaling mediated by ActRIIA in human placenta.

## Introduction

MicroRNA is an endogenous ∼22 nucleotides non-coding small RNA with repression of proteins by seed sequence pairing to the untranslating region (3′UTR) of target messenger RNAs [1]. According to the prevailing model, microRNA can direct gene down-regulation by two posttranscriptional mechanisms: mRNA cleavage or translational repression. The choice of posttranscriptional mechanism is determined by the identity of target. When 3′UTR of target mRNA has perfect complementary to microRNA, it will specify cleavage or it will repress the productive translation when it has imperfect complementary [2]. Controversially, some microRNAs have been reported that target mRNA also decreased in despite of imperfect complementary to 3′UTR of target mRNA [3], [4]. It is well known that microRNA can regulate almost one third of human genes [5]. In human, approximately one third of microRNAs are organized into clusters and it possibly leads to synergy in biological effects [6]. As microRNAs not only play important roles in cellular activities including cell proliferation, apoptosis, and cell fate determination [7], but also participate in many vital processes, such as immune responses, viral replications [8], [9]. The large regulatory network formulated by microRNAs makes them become the focus of researchers.

Preeclampsia is a syndrome manifested by on-set of hypertension and proteinuria after 20 weeks of gestation. It is the leading cause of maternal and fetal morbidity and mortality which has no good treatment except for delivery of the placenta [10]. In 2007, it was the first report that preelcampsia was associated with altered expression of microRNAs in placenta [11]. Later on, several other groups also published their similar findings in preeclamptic placentas [12]. It has been generally accepted that abnormal development of the placenta at early gestation may be the initial cause of the disease. Therefore, demonstration on the roles of the small molecules in human placentation will largely benefit clarifying the pathogenesis of preeclampsia.

MiR-195 is clustered with miR-497 [13] and belongs to miR-15 family [14]. It is well demonstrated that members of a microRNA family display overlapping, if not identical, roles, for sharing the same sequence binding to target mRNA [15], [16]. In human, miR-195 and mir-497 were shown potential tumor suppressor gene in primary peritoneal tumorgenesis [13]. Also, it is reported that miR-195 serves as tumor suppressor gene in colorectal cancer [17] and hepatocellular carcinoma [18]. In Xenopus, miR-15/miR-16 regulates early embryonic patterning by targeting Acvr2a (also named as ActRIIA), which is the type II receptor for ActivinA and Nodal [19]. When we performed bioinformatic analysis to predict target genes for miR-195 using four different programs, miRanda, TargetScan, miRBase, and PicTar, we found ActRIIA is one of the commonly predicted targets. It is well demonstrated that Activin/Nodal signaling plays substantial roles in the growth and differentiation of placental trophoblasts [20], [21], [22]. Ours and other’s studies have revealed up-regulation of ActivinA and Nodal in preeclamptic placenta, indicating involvement of abnormal ActivinA/Nodal signaling in pathogenesis of preeclampsia.

Based on above evidences, we hypothesized that miR-195 may participate in the regulation of placental trophoblast cell functions by targeting ActRIIA. To attest the hypothesis, we checked the direct binding of miR-195 to ActRIIA mRNA by using luciferase report assay in human placental trophoblast cell line, HTR8/Svneo cells. We further detected the influence of miR-195 on trophoblast cell invasion, and determined the rescue effect of ActRIIA on miR-195 function. The expression pattern of miR-195 and ActRIIA in preeclamptic placenta was further examined. The findings in the present study revealed that miR-195 could regulate trophoblast cells invasion via repressing ActRIIA protein expression, and down-regulation of the small molecule may be involved in the aberrant placenta function which contributes to the pathology of preeclampsia.

## Materials and Methods

### Patients

Placenta tissues from 17 normal pregnant women and 15 severe preeclamptic patients were collected at the Department of Obstetrics and Gynaecology, Peking University First Hospital, China. The study was approved by the Research Ethic Committees in Institute of Zoology, Chinese Academy of Sciences and in Peking University First Hospital. Written consents were obtained from all patients. Normal or uncomplicated pregnancy was defined as a unifetal gestation in a previously normotensive woman who did not suffer from high blood pressure and proteinuria during pregnancy, and delivered a healthy neonate with a weight adequate for gestational age after 37 weeks of pregnancy. Severe preeclampsia was defined according to the International Society for the Study of Hypertension in Pregnancy. In brief, these patients had no history of preexisting or chronic hypertension, but showed systolic blood pressure ≥160 mmHg or diastolic blood pressure ≥110 mmHg on at least 2 occasions, accompanying by significant proteinuria (>2 g/24 h or 3+ by dipstick in two random samples collected at >4 h interval) after 20 weeks of gestation. Those who developed renal disease, transient hypertension in pregnancy, gestational diabetes, spontaneous abortion, intrauterine fetal death, fetal chromosomal or congenital abnormalities or pregnancies conceived by fertility treatment were excluded from this study. The clinical characteristics of the patients included in this study were summarized in Table 1.

**Table 1:** Clinical characteristics of the pregnant women enrolled in this study.

	Normal pregnancy(n = 17)	Preeclampsia(n = 15)	*p*-value
Maternal age (years)	29.7±2.6	27.5±4.3	0.189
BMI (kg/m^2^)^a^	24.8±2.6	24.2±2.5	0.762
Systolic blood pressure (mmHg)	110.3±7.7	147.9±19.9*	<0.001
Diastolic blood pressure (mmHg)	71.8±5.6	95.3±16.5*	<0.001
50 g GCT (mmol/L)^b^	7.2±0.9	6.6±0.5	0.121
24 h urine protein (g)	0.03±0.06	4.16±2.72*	<0.001
Primiparous percentage (%)	71.4	60	NA.
Gestational day at delivery (Day)	272±9	262±18	0.071
Infant birth weight (g)	3517±468	2281±774*	0.004

Data are shown as Mean ±SEM, and significant difference between groups was analyzed with one-way ANOVA. *, compared with Normal pregnancy, *p*<0.05.

a. BMI, body mass index, indicating the weight in kilograms divided by the square of the height in meters.

b. GCT, glucose challenge test.

### Sequences and Constructs

The siRNA duplex against human ActRIIA, mature microRNA mimics for miR-195, miR-195 inhibitor and the scramble control were designed and purchased from Shanghai GenePharma, China. The sequences of siRNA and miRNA are shown in Table 2.

**Table 2:** The sequences of siRNA and miRNA.

Mimics	Sense	Anti-sense
Scramble control	UUCUCCGAACGUGUCACGUTT	ACGUGACACGUUCGGAGAATT
Si-ActRIIA	CAAUCAAACUGGUGUUGAATT	UUCAACACCAGUUUGAUUGGT
hsa-miR-195	UAGCAGCACAGAAAUAUUGGC	CAAUAUUUCUGUGCUGCUAUU
Anti-miR-195	GCCAAUAUUUCUGUGCUGCUA	

To construct the ActRIIA expressing plasmid (pcDNA4-ActRIIA), the coding sequence of ActRIIA was amplified and inserted into pcDNA4.0 vector (Invitrogen, Carlsbad, CA) at the EcoRI and HindIII restriction sites.

To generate pMIR-REPORT Luciferase plasmids for ActRIIA, 3′UTR segments of human ActRIIA mRNA (1694–1890 nt and 2010–2206 nt, Genbank accession no. NM_001616) containing the two putative miR-195 binding sequences were amplified and cloned into pMIR-REPORT Luciferase plasmid (Ambion, Austin, Texas, USA) at the Sac I and Hind III sites. The constructs were named as BD1-WT and BD2-WT. The mutated pMIR-REPORT plasmids, which carry site mutations in the 3′UTR segments of human ActRIIA mRNA being complementary to the seed sequence of miR-195, were generated based on BD1-WT/BD2-WT plasmids using QuikChange Lightning Site-Directed Mutagenesis Kit according the manufacture’s instruction (Stratagene, La Jolla, California, USA). The constructs were named as BD1-MUT and BD2-MUT. The primers for vectors construction are shown in Table 3. All the constructs were confirmed by DNA sequencing.

**Table 3:** Sequences of the primers used for plasmid construction or real-time PCR.

Product	Forward Primer	Reverse Primer
ActRIIA BD1 construct	GAGCTC ATGGTTGCGCCATCTGTG	AAGCTTATGTCCTTCACATCTGTGCT
ActRIIA BD2 construct	GAGCTC GGGTAACTTGTTTTTATTGCA	AAGCTTGCAACCGTGGAACTGAGGTAT
ActRIIA BD1-MUT construct	ACTCTGAACTGGAGCTGGTAAGCTAAAGAAACTGC	GCAGTTTCTTTAGCTTACCAGCTCCAGTTCAGAGT
ActRIIA BD2-MUT construct	CTTGTGAATGTTTAGTGTGCAGCTGTTCTGTGTACATAAAG	CTTTATGTACACAGAACAGCTGCACACTAAACATTCACAAG
ActRIIA expressing plasmid	AAGCTTATGGGAGCTGCTGCAAAGTT	GGATCCTAGCTTAGCAGCTCCAGTTCA
ActRIIA real-time PCR	AGGAGGAAATTGGCCAGCAT	GCGTCGTGATCCCAACATTC
GAPDH real-time PCR	AAGGTCATCCCTGAGCTGAAC	ACGCCTGCTTCACCACCTTCT
has-miR-195 real-time PCR	TAGCAGCACAGAAATATTGGC	
U6 real-time PCR	CGCAAGGATGACACGCAAATTC	

### Culture and Treatment of Trophoblast Cells

The immortalized human trophoblast cell line, HTR8/SVneo, was a kind gift from Dr. CH Graham at Queen’s University, Canada [23]. After thawing, the cells were maintained in RPMI1640 medium (Invitrogen, Carlsbad, CA) supplemented with 10% fetal bovine serum (complete medium), and were passaged at a ratio of 1∶5 every 3 days.

For transient transfection experiments, the cells were seeded in 6-well plates at 2*10^5^ cells per well in complete medium. Twenty-four hours after seeding, miR-195 mimics, inhibitors for miR-195, specific siRNA for ActRIIA, or pcDNA4/pcDNA4-ActRIIA was transfected into the cells using lipofectamine 2000 reagent according to the manufacturer’s instruction (Invitrogen, Carlsbad, CA). Six hours after transfection, the cells were recovered in the complete medium. The cells were subjected to further analysis at certain time as described below.

### RNA Extraction and Real-time PCR

Total RNA was extracted using TRIzol reagent (Invitrogen, Carlsbad, CA) following the manufacturer’s instruction. Two micrograms of total RNA were reverse-transcribed into cDNA using oligo (dT) primer (Tiangen Biotech Co, Beijing, China) and Moloney murine leukemia virus reverse transcriptase (Promega Corporation, Madison, USA). Reverse transcription of miRNA was performed according to the instruction of MiRcute MiRNA First-strand cDNA Synthesis Kit (Tiangen Biotech). In brief, poly(A) was added to 3′end of microRNA, and then oligo(dT)-universal tag was used as specific primer to generate cDNA for miRNA.

Real-time PCR was carried out using Applied Biosystems 7500 (Life Technologies, California, U.S.A), and the reaction mixture contained SYBR Green for cDNA (Takara Biotechnology Co, Dalian, China) or MiRcute MiRNA Premix for miRNA cDNA (Tiangen Biotech). The relative expression level of the detected genes was normalized against the value of corresponding GAPDH or U6. The sequences of primers are shown in Table 3.

### Protein Extraction and Western Blotting Analysis

Whole-cell lysates were prepared as described previously [22]. Protein samples were subjected to SDS-PAGE and transferred to nitrocellulose membrane. The membranes were incubated with 5% nonfat milk in PBST for 2 hours, and were then incubated with various primary antibodies overnight at 4°C. After washing in PBST, the membranes were subsequently incubated with horseradish peroxidase-conjugated secondary antibody (Jackson, PA, USA) for 2 hours. The antibodies used included goat anti-human ActRIIA (R&D, Minneapolis, USA), rabbit anti-human CCND1 (Abcam, Cambridge, UK), mouse anti-human CCND3 (Cell Signaling Technology, Beverly, MA, USA), mouse anti-human Actin (Cell Signaling Technology, Beverly, MA, USA), and mouse anti-human GAPDH (Ambion, Austin, Texas, USA). Signals were detected using an Enhanced Chemiluminescence Plus kit (Thermo Scientific, Rockford, USA) and visualized after exposure to a Kodak film. The membranes were scanned and signal intensities were analyzed by the Gel-Pro Analyzer (software version 4.0; United Bio). The relative densities of the detected molecules were measured by comparing their densitometry values with those of GAPDH in the same blot.

### Dual Luciferase Assay

HTR8/SVneo cells were plated into 24-well plates at a density of 5*10^4^ cells/well and transfected using Lipofectamine 2000 with 80 ng of pMIR-REPORT plasmid construct, 8 ng of pRL-TK control vector (encoding Renilla luciferase), and 20 nM of miR-195 in a final volume of 0.25 ml. Six hours after transfection, the cells were recovered in complete medium for another 18 hours. Cells were harvested 48 h post-transfection and luciferase activity was measured using the Dual-Glo luciferase assay system according to the manufacturer’s instructions (Promega, Madison, Wisconsin, USA). Each experiment was repeated for at least three times with triplicate in each group.

### Transwell Insert Invasion Assay

Transwell insert invasion assay was conducted in 24-well fitted inserts with membranes (8 µm proe size; Costar, Cambridge, MA, USA) as reported previously [24]. Briefly, 48 hours after transfection, cells were treated with 10 µg/ml mitomycineC for another 2 hours. Then the cells were trypsinized and seeded into transwell insert pre-coated with 200 µg/ml matrigel (BD Biosciences, USA) at 1*10^5^ cells per insert. The inserts contained RPMI 1640 medium (Invitrogen, Carlsbad, CA) plus 0.5% fetal bovine serum, and the lower chambers were loaded with RPMI1640 medium plus 10% fetal bovine serum. Thirty hours later, the cells were fixed and stained with hematoxylin. Non-invaded cells on the upper surface of the membrane were removed using a cotton swab. The number of stained cells at the lower surface of the membrane was counted in at least 15 randomly selected non-overlapping fields under light microscope. All experiments were done in triplicate and the invasion index was expressed as the percentage of invaded cell number compared with the corresponding control.

### MTT Assay

At certain time after seeding, cells were incubated in 1 mg/ml MTT solution (3-[4,5-dimethylthiazol-2-yl]-2,5-diphenyltetrazolium bromides; Sigma) for 4 h. After removal of MTT solution, dimethyl sulfoxide (DMSO) was added, and the absorbance at a wavelength of 490 nm (reference wavelength 690 nm) was recorded with Microplate Reader (Bio-Tek Instruments,Inc.). All experiments were done in triplicate.

### Gelatin Zymography

The presence of matrix metalloproteinase (MMP)-2 and MMP-9 in the media was demonstrated by gelatin zymography. The harvested culture media were standardized according to the protein contents of cell lysates. Ten to twenty microliter media were subjected to 10% SDS-PAGE containing 1 mg/ml gelatin. After electrophoresis, the gel was washed at room temperature for 1 h in 2.5% triton X-100, 50 mM Tris -HCl (pH7.5), and then incubated at 37°C overnight in a buffer containing 150 mM NaCl, 5 mMCaCl_2_ and 50 mM Tris-HCl (pH7.6). The gel was subsequently stained with 0.1%(w/v) Coomassie Brilliant Blue R-250, and destained in 10%(v/v) methanol and 5%(v/v) glacial acetic acid. The results were analyzed using Quantity One v4.6.2 software (Bio-Rad). All zymography experiments were repeated at least three times in duplicate.

### Statistical Analysis

Data of Transwell insert invasion assay, Dual luciferase assay, Real-time PCR and Western blotting were reported as Mean ± SEM according to the results of at least three independently repeated experiments. The statistical analysis was conducted by SPSS 17.0 software, and Least Significant Differences (LSD) method was performed as post-hoc analysis after one-way ANOVA to detect the significant differences. Differences were considered significant at *P*<0.05.

## Results

### ActRIIA Protein and miR-195 Exhibit Reversed Expression Pattern in Placentas from Severe Preeclamptic Patients

**Figure 1:**
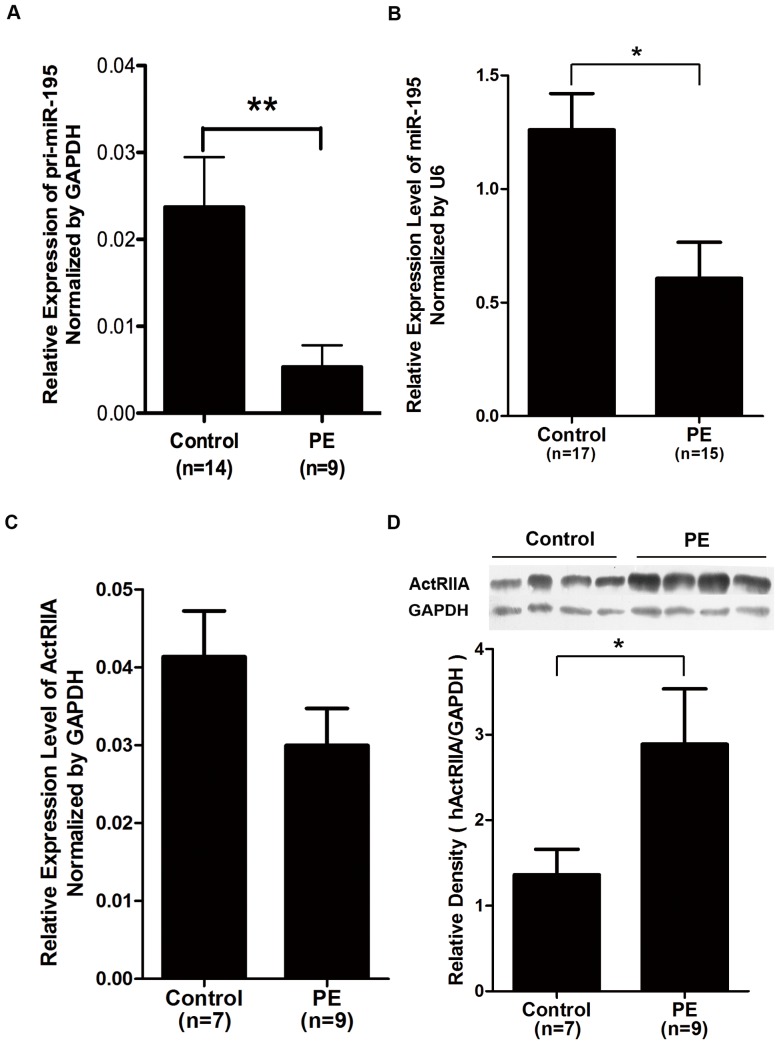
Expression of miR-195 and ActRIIA in placentas derived from severe preeclamptic patients (PE) and gestational-week-matched normal pregnant women (Control). A, B and C, Quantitative real time PCR to reveal pri-miR-195 (A), mature miR-195 (B) levels and mRNA expression of ActRIIA (C) in placentas from PE and Control. *, **, compared with Control, *p*<0.05, *p*<0.01. D, Western blotting to show protein levels of ActRIIA in placentas from PE and Control. Upper panel, a typical result of Western blotting. Lower panel, bar chart according to the statistical analysis based on the result of three independently repeat experiments. *, compared with Control, *p*<0.05.

We compared expression levels of pri-miR-195, miR-195 and ActRIIA in placentas from 15 severe preeclamptic patients and 17 normal pregnant women. There are no significant differences in age, body mass index (BMI), glucose tolerance (indicated by 50 g glucose challenge test), infant birth weight, and primiparous percentage between the normal pregnant and the preeclamptic women used in this study. As shown in Fig. 1A and 1B, relative expression of pri-miR-195 and miR-195 in PE placentas was significantly down-regulated to about 20% and 50%, respectively, of that in normal placentas. ActRIIA mRNA expression was measured by real-time PCR, and no obviously different relative level was observed between PE and normal placentas (Fig. 1C). Western blotting was performed to examine ActRIIA protein level, and two common samples were included in every blot as inner control to adjust variations in different blots. Statistic results showed that the relative density of ActRIIA in PE placentas was approximately 2-fold higher than that in controls (Fig. 1D).

### Validation of ActRIIA as the Direct Target of miR-195 in Human Trophoblast Cells

**Figure 2:**
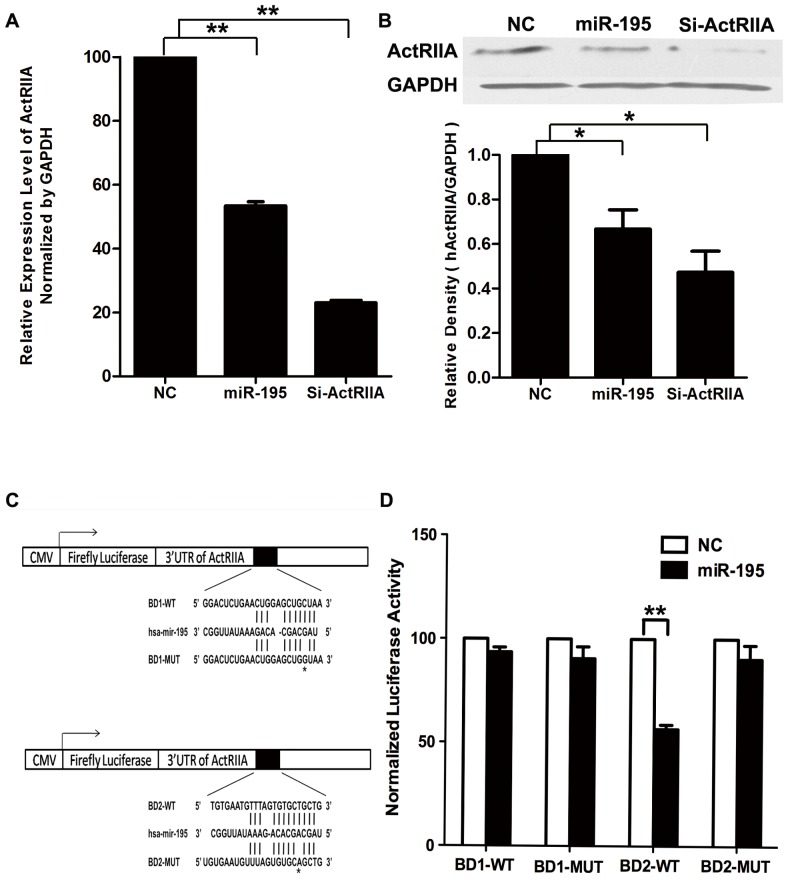
Validation of ActRIIA as target gene of miR-195 in HTR8/SVneo cells. A. Real-time PCR to reveal change of ActRIIA mRNA level in HTR8/SVneo cells transfected with scramble siRNA (NC), miR-195 mimics (miR-195) or ActRIIA siRNA (si-ActRIIA). **, compared with NC, P<0.01. B. Western blot analysis to show change of ActRIIA protein level in HTR8/SVneo cells transfected with NC, miR-195 or si-ActRIIA. Upper panel, typical results of Western blotting; Lower panel, bar chart representing the statistical analysis by ANOVA according to three independent experiments. The density of ActRIIA was adjusted by that of GAPDH in the same blot, and the values were presented as Mean±SEM. *, compared with NC, *p*<0.05. C. Schematic map for generating luciferase assay constructs. The constructs containing the region complementary to the seed sequence for miR-195 in 3′UTR segment of human ActRIIA gene are shown as BD1-WT and BD2-WT, and the mutant constructs are shown as BD1-MUT and BD2-MUT with asterisks indicating the mutation sites. D. Luciferase assay in HTR8/SVneo cells transfected with BD1-WT, BD2-WT, BD1-MUT or BD2-MUT reporter construct together with miR-195 mimics (miR-195) or scramble siRNA (NC). The value of corresponding control group (NC) was set as 100, and the results were presented as Mean±SEM according to three independent experiments. **, compared with corresponding NC, *p*<0.01.

As shown in Fig. 2A and 2B, transfection of miR-195 mimics in HTR8/SVneo cells resulted in down-regulation of ActRIIA expression at both mRNA and protein levels. The mRNA and protein levels of ActRIIA decreased to about 50% and 60% of controls that were transfected with scramble control.

According to the bioinformatic analysis, miR-195 has two binding sites in 3′UTR of ActRIIA mRNA. The seed sequence of miR-195 was complementary to the 47–53 nt or 502–508 nt of 3′UTR in ActRIIA mRNA. As shown in Fig. 2C, we generated two luciferase reporter constructs by cloning a 196 bp DNA fragment including binding site 1 (BD1) or binding site 2 (BD2)of the 3′UTR in human ActRIIA mRNA at downstream of the firefly luciferase reporter gene (named as BD1-WT and BD2-WT). A point-mutation was incorporated into the binding sites of 3′UTR in ActRIIA gene and the reporter constructs were named as BD1-MUT and BD2-MUT. HTR8/SVneo cells were transfected with BD1-WT/BD2-WT or BD1-MUT/BD2-MUT reporter construct together with miR-195 mimics as well as renilla luciferase vector (pRL-TK) to be used as reference control. Relative luciferase activity was monitored 48 h after transfection. As shown in Fig. 2D, miR-195 mimics could evidently reduce the relative luciferase activity of BD2-WT construct by about 45% compared with scramble control, whereas did not show any suppression on that of BD2-MUT construct. However, miR-195 mimics could not affect the relative luciferase activity of BD1-WT/BD1-MUT construct. The data strongly validated ActRIIA as target gene of miR-195 in human trophoblast cells, and the 502–508 nt of 3′UTR in ActRIIA gene was the real binding site for miR-195.

### Effects of miR-195 on Cell Invasion in HTR8/SVneo Cells

To further demonstrate the influence of miR-195 on trophpoblast cell behaviors, we examined cell proliferation and invasion in HTR8/SVneo cells that were transfected with miR-195 or inhibitor of the small molecule.

**Figure 3:**
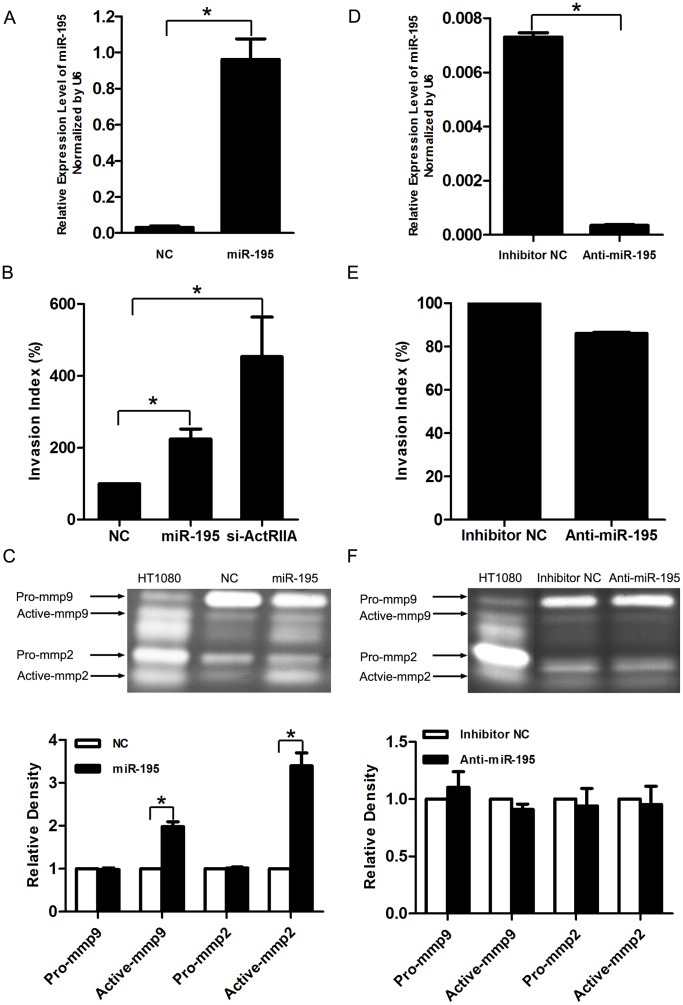
Effect of miR-195 on cell invasion in HTR8/SVneo cells. A and D. The expression of miR-195 in HTR8/SVneo cells transfected with miR-195 mimics (miR-195), scramble siRNA (NC), miR-195 antigomir (Anti-miR-195) or scramble antigomir (Inhibitor NC) was measured by real time PCR. The level of miR-195 was adjusted by that of U6, and the relative value was presented as Mean±SEM according to three independent experiments. *, compared with corresponding NC, *p*<0.05. B and E. Transwell insert assay to examine cell invasiveness transfected with NC, miR-195, siRNA for ActRIIA (si-ActRIIA), Inhibitor NC or Anti-miR-195. The value of invasion index in corresponding NC was set as 100, and the results were presented as Mean±SEM according to three independent experiments. *, compared with corresponding NC, *p*<0.05. C and F. Gelatin zymography to measure MMP-2 and MMP-9 productions in cells transfected with miR-195, NC, Inhibitor NC or Anti-miR-195. Upper panels, typical results of gelatin zymography. Conditioned media of HT1080 cells was included as positive control for zymography. The pro- and active forms of MMP-2 and MMP-9 were separately indicated. Lower panels, bar charts representing the statistical analysis by ANOVA according to three independent experiments. The densities of pro- and active forms of MMP-2 and MMP-9 were presented as Mean±SEM. *, compared with data in corresponding NC, *p*<0.05.

As shown in Fig. 3A and D, transfection of mimics or inhibitors for miR-195 could significantly increase or repress the relative expression of miR-195 in HTR8/SVneo cells. In these cells, neither mimics nor inhibitors for miR-195 changed cell viability as measured by MTT assay (data not shown). Even so, to avoid the possible influence of cell growth on the result of cell invasion, we treated the cells with mitomycinC when performing transwell insert invasion assay. Data revealed that overexpression of miR-195 in HTR8/SVneo cells evidently promoted cell invasiveness as well as the activation of MMP-9 and MMP-2, which have been shown to be the most important MMPs involved in trophoblast cell invasion (Fig. 3B and C). However, inhibition of miR-195 did not affect cell invasiveness and MMP-9 or MMP-2 activation. We assume that the endogenous level of miR-195 in HTR8/SVneo cells is too low to exhibit significant impact on cell invasion (Fig. 3E and F).

### Overexpression of ActRIIA Attenuates the Invasion-promoting Effect of miR-195 in HTR8/SVneo Cells

**Figure 4:**
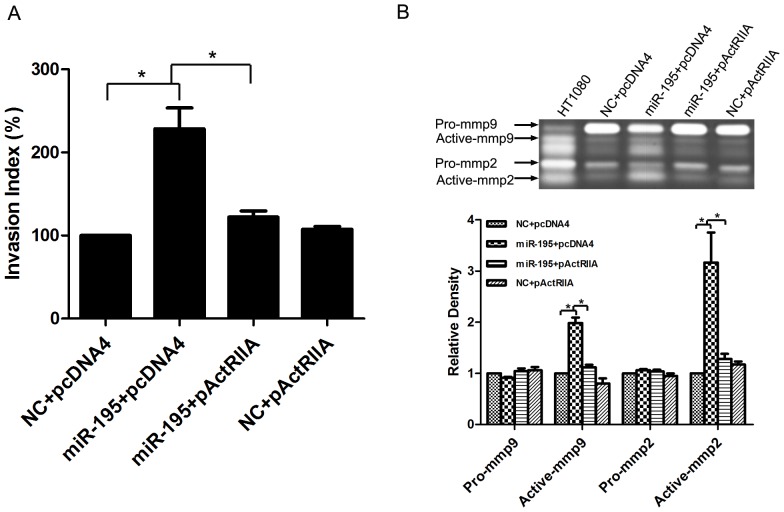
ActRIIA rescued the effect of miR-195 on cell invasion in HTR8/SVneo cells. HTR8/SVneo cells were transfected with miR-195 and ActRIIA alone or in combination, with scramble siRNA (NC) or pcDNA4 vector (pcDNA4) as corresponding negative control. Transwell insert assay was performed to monitor cell invasiveness (A) and Gelatin zymography was performed to measure MMP-2 and MMP-9 productions (B). The statistical analysis was carried out by ANOVA according to three independent experiments, and the values were presented as Mean±SEM. *, compared with corresponding group as indicated, *p*<0.05.

We then tried to figure out whether ActRIIA directly participated in the invasion-promoting effect of miR-195. Knockdown of ActRIIA by specific siRNA had similar invasion-promoting effect in HTR8/SVneo cells (Fig. 3B). We then performed a “rescue” experiment by transfecting the cells with miR-195 together with ActRIIA-expressing construct (pcDNA4-ActRIIA). It was shown that overexpression of ActRIIA could completely block the invasion-promotion as well as activation of MMP-9 and MMP-2 caused by miR-195 in HTR8/SVneo cells (Fig. 4A and B). The data demonstrated that ActRIIA was directly involved in the invasion-promoting effect of miR-195 in HTR8/SVneo cells.

### MiR-195 did Not Influence CCND1/3 Expression in HTR8/SVneo Cells

**Figure 5:**
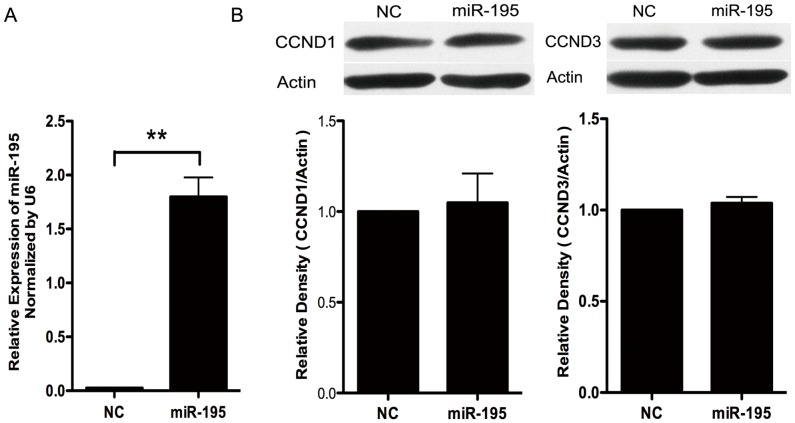
Effect of miR-195 on protein expression of CCND1 and CCND3 in HTR8/SVneo cells. A. Real-time PCR to reveal miR-195 level in HTR8/SVneo cells transfected with scramble siRNA (NC) or miR-195 mimics (miR-195). **, compared with NC, *p*<0.01. B. Western blot analysis to show change of CCND1 and CCND3 protein level in HTR8/SVneo cells transfected with NC or miR-195. Upper panels, typical results of Western blotting; Lower panels, bar charts representing the statistical analysis by ANOVA according to three independent experiments. The densities of CCND1 and CCND3 were adjusted by that of Actin in the same blot, and the values were presented as Mean±SEM.

Literature reports revealed that miR-195 could inhibit cell invasion by inhibiting CCND1 and CCND3 in breast cancer cells and gliobalstoma cells. We therefore examined CCND1 and CCND3 expression in HTR8/SVneo cells transfected with miR-195. As shown in Figure 5, no change in protein levels of CCND1 and CCND3 was observed in HTR8/SVneo cells at 48 h after miR-195 transfection.

## Discussion

MicroRNAs are newly identified modulators of many complicated signaling pathways involved in various human diseases. In preeclamptic placentas, several reports demonstrated differentially expressed microRNAs compared with normal pregnant ones [11], [12], [25]. MiR-210 and miR-155 have been subjected to further functional studies, and data revealed that they are related to angiogenesis and trophoblast cell invasion/migration [26], [27], [28]. By far, it remains to be clarified as to how the deregulated miRNAs participate in the occurrence of preeclampsia.

MiR-195 is one of the differential miRNAs in preeclamptic placentas reported by Zhu et al. [12]. Here we confirmed the down-regulation of this small molecule in preeclamptic placentas from Chinese Han women. By far, functional studies on miR-195 are mainly in cancer, whereas little is known about its role in placenta. This small molecule attracted our attention for three reasons: (i) the target prediction result shows that ActRIIA is one of its possible targets at high value. As we know, ActRIIA is the type II receptor for Activin and Nodal, and Activin/Nodal signaling has been shown to play critical roles in modulating functions of placental trophoblast cells and endothelial cells during pregnancy. (ii) There are two predicted miR-195 binding sites in 3′UTR of ActRIIA mRNA, and the binding sites are evolutionarily conserved from amphibians to humans. This suggests that modulation of ActRIIA by miR-195 may be biologically conserved during evoluation. (iii) Our previous work and other’s reports demonstrated overexpression of ActivinA and Nodal in preeclamptic placenta, indicating excessive activation of the signaling during occurrence of the pregnancy disorder [29]. Our data revealed that the expression of miR-195 was in an inverse association with protein level of ActRIIA in severe preeclamptic placentas, indicating that miR-195 may play roles in the excessive activation Nodal/ActivinA signaling observed in preeclamptic placenta.

MiR-195 belongs to miR-15 gene family which includes miR-15a/b/c, miR-16a/b/c, miR-497 and miR-424. In mamanlian, only miR-195, miR-497 and miR-424 are expressed [30]. From literatures of microarray data, miR-195, miR-15b and miR-424 were the three members in miR-15 gene family that exhibited down-regulation in preeclamptic placenta [12], [31]. Functional studies of these small RNAs in cancer cells revealed that they mainly participated in regulating cell apoptosis, proliferation and invasion. For instance, miR-195 could control cell cycle through targeting cyclin D1, CDK6, and E2F3, and thus suppress the ability of HCC cells to form colonies in vitro and to develop tumors in nude mice [18]. MiR-195 could also promote colorectal cancer cell apoptosis and suppress tumorigenicity by targeting BCL-2 [17]. Recently, miR-195 and miR-497 were shown to suppress breast cancer cell proliferation and invasion via targeting Raf-1 and CCND1 [32]. In glioblastoma cells, miR-195 could inhibit cell invasion by inhibiting CCND3 and cause cytoplasmic accumulation of p27 [33]. MiR-15b could regulate cell cycle progression in glioma cells by targeting cyclin E1 [34]. MiR-424 affected human dermal microvascular ECs (HDMECs) proliferation via MEK1 and cyclin E1 [35]. Human placental trophoblast cells have similar properties as tumor cells in the aspects of active cell proliferation and invasion, although their behaviors are temporally and spatially restricted during pregnancy. In human trophoblast cell line, HTR8/SVneo cells, no change in cell number is observed after miR-195 transfection. This is different from the observations in hepatocellular carcinoma cells and colorectal cancer cells [17], [18]. Meanwhile, miR-195 shows strongly invasion-promoting effect in HTR8/SVneo cells, which is absolutely opposite to the results observed in breast cancer and glioblastoma cells [32], [33]. The two experimentally proved targets involved in invasion-inhibiting effect of miR-195 in cancer cells, CCND1 and CCND3, are not influenced by miR-195 in human trophoblastic HTR8/SVneo cells. Therefore, it is most likely that miR-195 works in a unique way in human trophoblast cells which is different from what it does in cancer cells. Considering that the differentiation status and properties of trophoblast cells vary along gestation, the roles of miR-195 should be tightly associated with placental developmental stages. Therefore, further detailed studies on the localization and expression patterns of miR-195 in human placenta along the whole gestation are necessary for clarifying its working mechanisms.

Targets exploration is critical for clarifying the mechanism of microRNAs. Based on the results of target prediction and expression pattern of miR-195 in preeclamptic placentas, we further evaluate whether ActRIIA is functionally involved in trophoblast cell invasion regulated by miR-195. Several lines of evidence prove that ActRIIA is a target of miR-195 in human trophoblasts. First, ActRIIA expression is suppressed by miR-195 in HTR8/SVneo cells. Second, the regulation of miR-195 on ActRIIA expression is likely direct, because the luciferase report construct containing the 3′UTR region of ActRIIA mRNA with the putative miR-195 binding sequence is specifically responsive to miR-195 overexpression by inhibition on the luciferase activity. There are two putative conserved binding sites in the 3′UTR region of ActRIIA mRNA, but only one of them is proved to be directly responsive for miR-195 in human trophoblast cells. Third, the inhibitory effect of miR-195 on the luciferase activity is abolished upon mutation in the binding sites paired by the seed sequence of miR-195. More importantly, ActRIIA could substantially abrogate the effect of miR-195 on cell invasion and activation of MMP-2 and MMP-9 in HTR8/SVneo cells. These findings indubitably indicated that ActRIIA is one of the critical targets, at least, in mediating the invasion-promoting role of miR-195 in human trophoblast cells. It is noticeable that miR-15/16 restrict the size of the Spemann’s organizer by targeting ActRIIA during embryo development in Xenopus [19]. The binding sites in ActRIIA mRNA for miR-15/16 and miR-195 are conserved, further suggesting that ActRIIA may be an essential target for these members of miR-15 family.

In human placenta, ActRIIA is mainly localized in trophoblast cells at the first and second trimester, and extensive in vascular endothelium at term pregnancy [36] It’s well known that ActRIIA is common type II receptor for ActivinA and Nodal. The two ligands bind to different type I receptor (ALK4 or ALK7) [37], and cause opposite effect on trophoblast cell invasion [20], [29]. The invasion-promoting effect of miR-195 observed in HTR8/SVneo cells most likely results from interference on Nodal signaling mediated by suppressed expression of ActRIIA, as it has been shown that Nodal inhibits, whereas ActivinA promotes trophoblast cell invasion. Our previous study has demonstrated up-regulation of Nodal expression in PE placenta [29]. The increase of ActRIIA receptor resulting from, at least in part, lowered miR-195 in PE placenta will lead to further augment of Nodal signaling and therefore inhibition on trophoblast cell invasion. Abnormal placental development, especially shallow invasion of trophoblast cells into decidual stroma and spiral arteries at early gestation has been generally accepted as the causal factor for the complex syndrome [38]. Our data regarding the expression pattern of miR-195 and ActRIIA in PE placenta as well as their regulation on trophoblast cell invasion are likely indicating the way whereby miR-195 participates in the pathogenesis of PE.

The mechanism of miR-195 down-regulation in preeclamptic placenta remains unknown. High frequency of loss of heterozygosity has been found on chromosome 17p13.1 where miR-195 located, which is implicated to play important roles in the pathogenesis of many types of tumors [39]. Recently, the methylation state of CpG islands upstream of the miR-195/497 gene was found to be responsible for the down-regulation of both miRNAs in breast cancer [32]. Our finding of lowered expression of pri-miR-195 in PE placenta strongly indicates the aberrant transcription of this small RNA during the pathological process. Whether allelic loss and promoter hypermethylation can account for the reduced miR-195 expression in PE placentas needs further investigation.

This study extends our knowledge on the functions of miR-195 in human placenta. The specifically aberrant regulation on miR-195 expression may contribute to the occurrence of preeclampsia through destroying physiological role of Nodal signaling in trophoblast cells. Given that a single microRNA can target many genes, we believe that miR-195 also has multiple targets. It will greatly help us to better understand the mechanism of miR-195 in human placentation after more functional targets are identified. In addition, a precise expression timing and localization of the small RNA and its target genes during placenta development need to be taken into account to identify their working mechanisms.
